# The effects of maternal depression on their perception of emotional and behavioral problems of their internationally adopted children

**DOI:** 10.1186/s13034-021-00396-0

**Published:** 2021-08-23

**Authors:** Krista Liskola, Hanna Raaska, Helena Lapinleimu, Jari Lipsanen, Jari Sinkkonen, Marko Elovainio

**Affiliations:** 1 Department of Child Psychiatry, Helsinki University Hospital and University of Helsinki, P.O. Box 590, 00029 Helsinki, Finland; 2 Department of Pediatrics and Adolescent Medicine, Turku University Hospital and University of Turku, Turku, Finland; 3 Department of Child Psychiatry, University of Turku, Turku, Finland; 4 Department of Psychology and Logopedics, University of Helsinki, Helsinki, Finland; 5 National Institute for Health and Welfare, Helsinki, Finland; 6 Research Program Unit, Faculty of Medicine, University of Helsinki, Helsinki, Finland

**Keywords:** Depression-distortion hypothesis, Adoption, CBCL, GHQ

## Abstract

**Background:**

Even though child psychopathology assessment guidelines emphasize comprehensive multi-method, multimodal, and multi-informant methodologies, maternal-report symptom-rating scales often serve as the predominant source of information. Research has shown that parental mood symptomatology affects their reports of their offspring’s psychopathology. For example, the depression-distortion hypothesis suggests that maternal depression promotes a negative bias in mothers’ perceptions of their children’s behavioral and emotional problems. We investigated this difference of perception between adoptive mothers and internationally adopted children. Most previous studies suffer from the potential bias caused by the fact that parents and children share genetic risks.

**Methods:**

Data were derived from the Finnish Adoption (FinAdo) survey study (a subsample of adopted children aged between 9 and 12 years, n = 222). The Child Behavior Checklist (CBCL) was used to assess emotional and behavioral problems and competences of the adopted children. The CBCL was filled in by the adopted children and the adoptive mothers, respectively. Maternal depressive symptoms were measured using the short version of the General Health Questionnaire.

**Results:**

On average, mothers reported less total CBCL symptoms in their children than the children themselves (0.25 vs 0.38, p-value < 0.01 for difference)*.* Mothers’ depressive symptoms moderated the discrepancy in reporting internalizing symptoms (β = − 0.14 and p-value 0.01 for interaction) and the total symptoms scores (β = − 0.22 and p-value < 0.001 for interaction) and externalizing symptoms in girls in the CBCL.

**Limitations:**

The major limitation of our study is its cross-sectional design and the fact that we only collected data in the form of questionnaires.

**Conclusions:**

The results of our research support the depression-distortion hypothesis concerning the association of maternal depressive symptoms and child internalizing symptoms and externalizing symptoms in girls in a sample without genetic bias

## Introduction

In the assessment of emotional and behavioral psychiatric symptoms in children, the procedure of collecting and combining information from multiple sources, e.g. mother, father, therapist, teacher, or foster parent, has become the standard of practice [36]. However, research has consistently shown that the agreement between ratings of child behavior by different informants is only low to moderate [14, 19, 34, 47] and considerable discrepancies have also been observed between parent and adolescent reports of adolescent behavior [15]. Furthermore, it has been argued that the nature of discrepancies differs by type of behavior [35].

A number of possible explanations have been given to this discrepancy. The depression-distortion hypothesis suggests that maternal depression promotes a negative bias in mothers’ perceptions of their children’s behavioral and emotional problems [13, 39, 40], with recent evidence indicating that this effect may be greater in questionnaires than in clinical interviews [32]. This hypothesis does not imply that depressed mothers perceive their children in a more negative way, but that their perception is biased. The Attribution Bias Context model (ABC model) suggests that reporting discrepancies may result from discordant perspectives between informants [15, 43]. In support of the ABC model and depression-distortion hypothesis, previous research has demonstrated that parents’ depression and anxiety are associated with over-reporting children’s problem behaviors [15, 20, 23, 46]. However, some research shows that when other variables, such as family functioning, are controlled, the impact of mood on ratings is non-significant or small [16, 49].

It has been argued, that children of psychiatrically ill mothers show more symptomatic behavior than do the children of healthy mothers [10, 37] at least partly due to shared genetic background. Maternal psychopathology appears therefore to be related to actual child psychopathology by means of genetic transmission, social learning processes, lack of maternal sensitivity, insecure attachment patterns or inadequate or inconsistent parenting behavior. Higher maternal ratings may therefore also reflect a truly higher level of mental health symptoms in their children. This alternative assumption has been called the accuracy model [33]. Although the combinatory model suggesting that maternal ratings of child psychiatric symptoms might be influenced simultaneously by pathologic distortions by the mother and by a truly increased level of child psychiatric symptoms may be reasonable, the evaluation of the relative effects of the two are difficult to detect when parents and children share genetic risks.

In this study, we investigated the influence of maternal psychopathology, more precisely depressive symptoms, on the rating of emotional and behavioral problems of their internationally adopted children. We tested the effect of maternal depressive symptoms on the difference between maternal and child ratings. Paternal ratings were excluded, as the number of fathers that filled in the questionnaires was low and the cohort included single mothers as well. Whereas most research on cross-informant agreement or discrepancy focuses on biological offspring, in our sample these mother–child couples are genetically unrelated.

We hypothesized that a discrepancy would also be observed between adoptive mothers and internationally adopted children.

## Methods

### Participants

This study is part of the ongoing FINnish ADOption (FinAdo) study. The target population of the study consists of all children internationally adopted through three legalized adoption organizations in Finland between 1985 and 2007. The children were identified through official adoption organizations approved by the Ministry of Social Affairs and Health. Data were gathered with questionnaires exploring information about the child, the adoptive family and the parents themselves. The questionnaires were sent to the parents and in the cover letter it was instructed that the child/adolescent fills in their own questionnaires and there was a separate return envelope for the child. The questionnaires were filled in separately by the parents and those adoptees over 9 years of age. The reason for the age of 9 years as the cut-off was the fact that they were the youngest that responded themselves to the questionnaires and thus comparing mothers’ and childs’ ratings was possible only for them and older participants. The average age at the time of estimation was 14.1 for boys and 13.9 for girls.

The surveys were conducted in December 2007, January 2008 and March 2009. The original cohort included 634 boys and 803 girls at study entry, aged from 0 to 18 years (mean 7.5, Sd 4.4). The study sample (n = 222) consisted of 124 girls (55.86%) and 98 boys (44.14%) and their mothers. Fathers were excluded, as the respondents were mainly the mothers. The characteristics of the sample are shown in Table 1.

**Table 1:** Characteristics of the sample

Characteristics	Mean (SD)	N (%)
Total number of children		222
Gender		
Boys		99 (44.14)
Girls		124 (55.86)
Age at arrival in Finland	3.7 (2.6)	
Boys	4.4 (2.8)	
Girls	3.2 (2.2)	
Age at time of estimation	14.0 (0.9)	
Boys	14.1 (0.9)	
Girls	13.9 (0.9)	
Continent of birth		
Asia		47 (21.17)
Europe		41 (8.46)
Africa		13 (5.86)
America		31 (13.96)
Not available		90 (40.54)
Type of placements before adoption		
Orphanage		74 (33.33)
Foster home		19 (8.56)
Multiple placements		129 (58.11)
Mother’s employment status		
Managerial employee		88 (39.6)
Clerical worker		40 (18.0)
Worker		80 (36.0)
Other		14 (6.3)
Mother’s age	37.86 (5.36)	

The study was approved by the Ethics committee of the Hospital district of Southwest Finland and written and informed consent was obtained from the parents and the children themselves.

## Measures

### Child-related background factors

A specific questionnaire developed for the FinAdo study was used to gain knowledge about the characteristics of the child before and after adoption. The child-related variables included the child’s gender, age at the time of adoption, which is the same as age at arrival to Finland, and at the time of responding to the questionnaire, continent of birth, the type and number of pre-adoption placements and health history. Maternal SES was defined as the mother’s employment status, which reflects maternal education. Occupational status, although not the most comprehensive measure of SES, is widely used e.g. [29, 31].

### Parental depressive symptoms

The General Health Questionnaire (GHQ) is a self-administered screening questionnaire, designed for use in consulting settings aimed at detecting individuals with a diagnosable psychiatric disorder [21]. The 12-Item General Health Questionnaire (GHQ-12) is the most extensively used screening instrument for common mental disorders, in addition to being a more general measure of psychiatric well-being. Various versions of the GHQ-12 have been reported to be useful in determining the presence of depression and also shorter (five item) versions have shown good predictive validity [1]. In this study, we used a five-item questionnaire requesting whether the parent had recently been able to enjoy his/her daily duties, been thinking of himself/herself as a worthless person, felt unhappy and depressed, lost his/her self-confidence, or felt quite happy. The questions were answered on a 4-point scale: 1 = more than usual, 2 = as much as usual, 3 = less than usual, 4 = much less than usual. The first and last items were reverse coded and all items were summed.

### The adopted children’s emotional and behavioral problems

The Child Behavior Checklist is a component in the Achenbach System of Empirically Based Assessment developed by Thomas M. Achenbach. It is a 118-question behavioral checklist that is completed by the child’s parent or caretaker. It is an instrument designed to obtain data on children’s behavioral/emotional problems and competencies, and it is widely used in clinical and research settings because of its demonstrated reliability and validity, ease of administration, and applicability to clinical and nonclinical groups [17]. In our study, we chose to use the CBCL to measure behavioral problems because of its wide use as a well-known method in adoption research and its good psychometric reliability [22, 26, 50].

The CBCL provides a total score for behavioral characteristics and separates scores for internalizing and externalizing behavioral symptoms. Internalizing behavioral symptoms reflect problems mainly within the self, such as anxiety, depression, somatic complaints without medical cause, and withdrawal from social contacts. Externalizing behavioral signs include conflict with others and rule-breaking or aggressive behavior [2, 3].

A high level of association between the CBCL and diagnoses derived via structured interviews has been documented. For example, studies have found associations between depressive disorders and the depression/anxiety, withdrawn, and somatic complaints subscales, as well as with the broadband internalizing scale. Similarly, anxiety disorders have been significantly associated with elevated scores on the depression/anxiety subscale. In addition, these studies have found significant associations between conduct disorder and the aggressive behavior, delinquent behavior, and the broadband externalizing subscales of the CBCL [7–9, 18, 28, 52].

We used the CBCL for ages 6–18 with 113 questions [22], leaving out the 5 open questions. Each item was rated as (0) not true, (1) somewhat or sometimes true, and (2) very true or often true. The higher the child’s scores in the CBCL, the more behavioral problems the child has.

The CBCL was filled in by the adoptive mothers and the adopted children, respectively.

### Statistical analyses

The associations between maternal depressive symptoms which were treated as continuous and the CBCL were analyzed using linear regression models with child CBCL reports as outcomes and mother CBCL reports as predictors. The interaction term between mothers’ depressive symptoms and their CBCL reports was added into the models to test the significance of the difference between with higher depressive symptoms and lower depressive symptoms reporting discrepancy.

All analyses were performed using R 3.6.1, and *p* values below 0.05 were considered statistically significant.

## Results

The mean level of depressive symptoms in mothers was 1.89 (SD 0.51). The mean score of externalizing symptoms was 0.28 (SD 0.32) for mothers and 0.38 (SD 0.30) for children. For internalizing symptoms, the mean scores were 0.19 (SD 0.20) for mothers and 0.33 (SD 0.28) for children. The mean CBCL total score for mothers was 0.25 (SD 0.24) and for children 0.38 (SD 025). Mothers’ depressive symptoms were not associated with their children’s CBCL symptoms reported by themselves or by their children (Table 2).

**Table 2:** Means, standard deviations, and correlations with confidence intervals

Variable	*M*	*SD*	1	**2**	**3**	4	**5**	6
1. Depression (mother)	1.89	0.51						
2. CBCL internal (mother)	0.19	0.20	0.12 [− 0.01, 0.25]					
3. CBCL external (mother)	0.28	0.32	0.07 [− 0.06, 0.20]	0.52** [0.41, 0.61]				
4. CBCL total (mother)	0.25	0.24	0.07 [− 0.06, 0.20]	0.83** [0.78, 0.87]	0.83** [0.78, 0.87]			
5. CBCL internal (self)	0.33	0.28	− 0.02 [− 0.15, 0.11]	0.35** [0.23, 0.46]	0.11 [− 0.02, 0.24]	0.26** [0.14, 0.38]		
6. CBCL external (self)	0.38	0.27	− 0.07 [− 0.20, 0.06]	0.23** [0.10, 0.35]	0.50** [0.40, 0.60]	0.38** [0.26, 0.49]	0.56** [0.46, 0.64]	
7. CBCL total (self)	0.38	0.25	− 0.04 [− 0.17, 0.09]	0.32** [0.20, 0.43]	0.26** [0.14, 0.38]	0.33** [0.21, 0.44]	0.92** [0.89, 0.93]	0.79** [0.74, 0.84]

*M* and *SD* are used to represent mean and standard deviation, respectively. Values in square brackets indicate the 95% confidence interval for each correlation. The confidence interval is a plausible range of population correlations that could have caused the sample correlation [12]

*Indicates *p* < 0.05. **Indicates *p* <0 .01

**Fig. 1:**
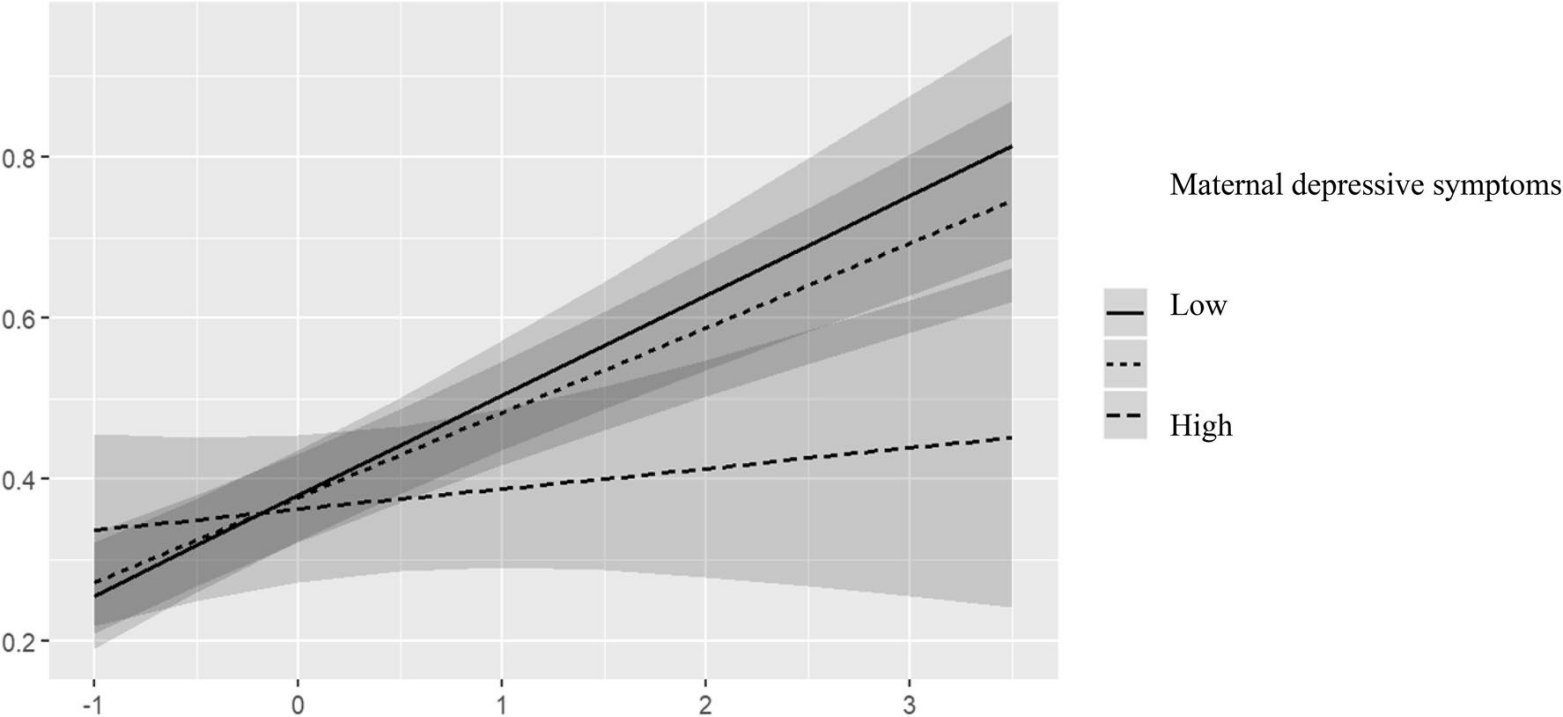
Interaction effect between mothers depressive symptoms (classified as high =  + 1 SD, and Low = − 1 SD) and mother-rated internalizing symptoms for child-rated internalizing symptoms (testing modifying effect of mothers depressive symptoms in mother and child-rated similarity in internalizing symptoms)

**Fig. 2:**
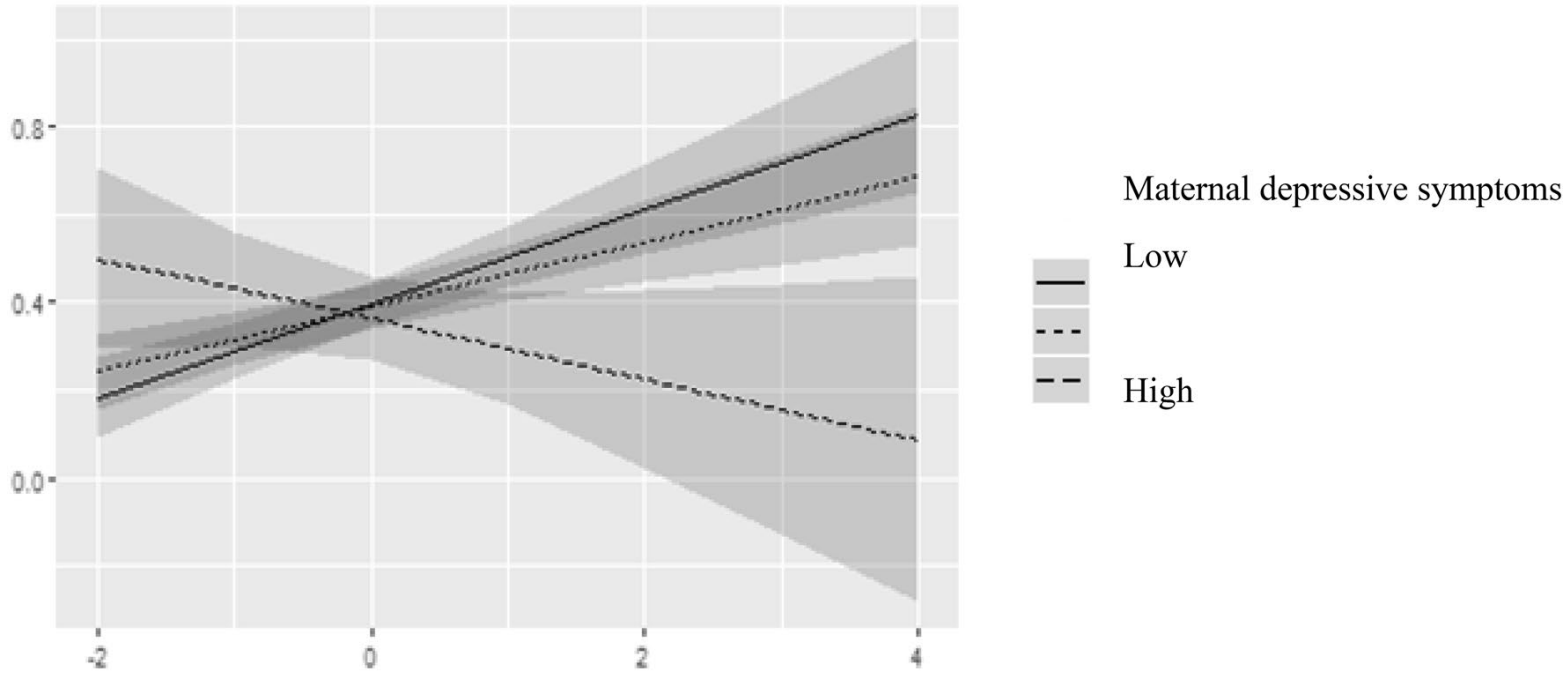
Interaction effect between mothers depressive symptoms (classified as high =  + 1 SD, and Low = − 1 SD) and mother-rated total CBCL symptoms for child-rated total CBCL symptoms (testing modifying effect of mothers depressive symptoms in mother and child-rated similarity in total CBCL symptoms)

**Fig. 3:**
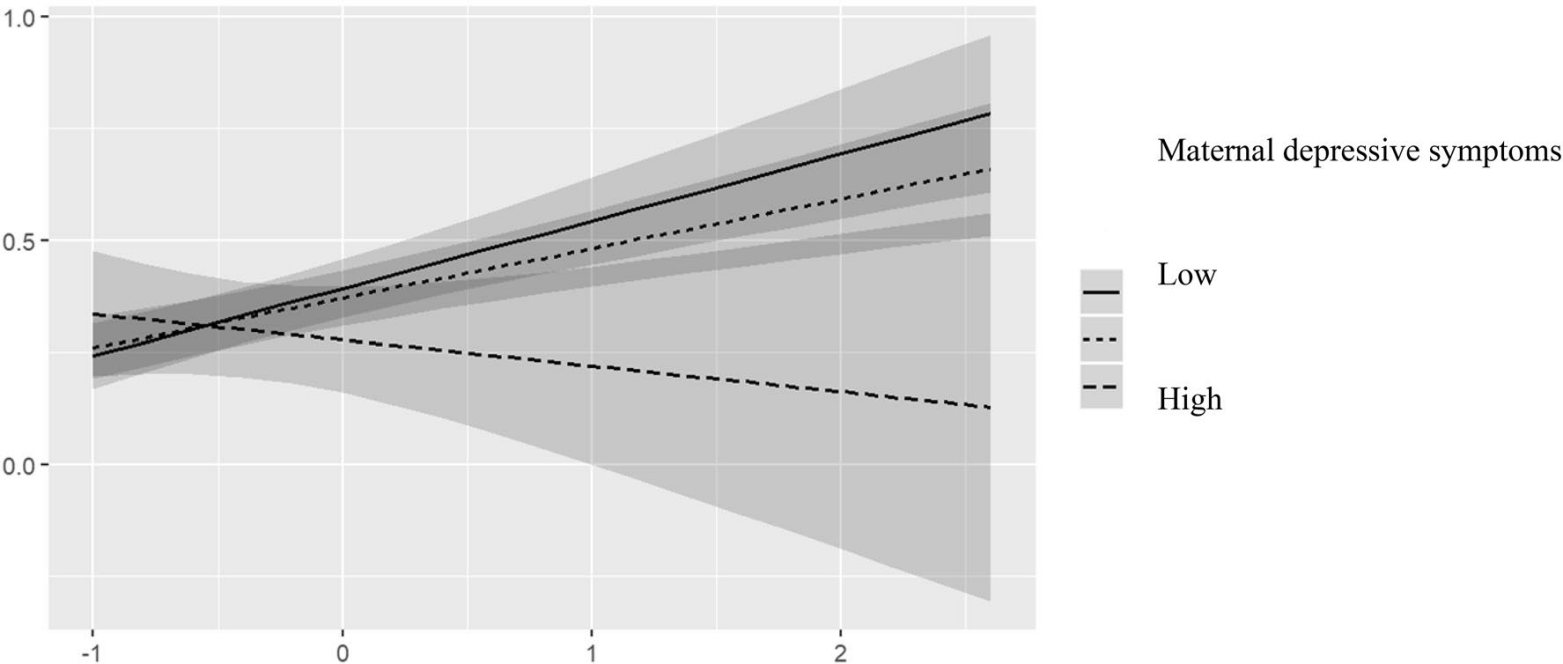
Interaction effect between mothers depressive symptoms (classified as high =  + 1 SD, and Low = − 1 SD) and mother-rated externalizing symptoms for child-rated externalizing symptoms (testing modifying effect of mothers depressive symptoms in mother and child-rated similarity in externalizing symptoms)

There were significant interaction effects between mothers’ depressive symptoms and mother reported internalizing symptoms (p-values for interaction range from (0.013 to 0.006) (Table 3). The higher the mothers depressive symptoms the less similar the mother and child-reported symptoms were (Fig. 1). Similar interaction effect was found between mothers’ depressive symptoms and mother reported total CBCL score (p-values range for interaction effect range from 0.010 to 0.001) (Table 4; Fig. 2). There were also significant interaction effects between mothers depressive symptoms and mother reported externalizing symptoms (p-values for interaction range from (0.028 to 0.005), but these effects were only evident in girls (third level gender interactions p-value range (0.025–0.011) (Table 5). Again, the more mother had depressive symptoms the less similar the symptom reporting was between the mother and the child (Fig. 3). All these models were adjusted for child’s age, age at arrival to Finland and mother’s SES.

**Table 3:** Interaction between mother-rated CBCL internalizing symptoms and maternal depression for child-rated CBCL internalizing symptoms

Predictors	CBCL internal (child)			CBCL external (child)			CBCL total (child)		
	Beta	95% CI	*p*	Beta	95% CI	*p*	Beta	95% CI	*p*
Depression (mother)	− 0.02	− 0.15 to 0.11	0.725	− 0.08	− 0.21 to 0.06	0.280	− 0.06	− 0.19 to 0.08	0.404
CBCL internal (parent)	0.36	0.24 to 0.48	** < 0.001**	0.27	0.14 to 0.40	** < 0.001**	0.34	0.22 to 0.47	** < 0.001**
Depression (mother) × CBCL internal (parent)	− 0.15	− 0.26 to − 0.04	**0.010**	− 0.15	− 0.27 to − 0.03	**0.013**	− 0.16	− 0.27 to − 0.04	**0.006**
Observations	222			222			222		
R^2^/R^2^ adjusted	0.210/0.176			0.128/0.091			0.178/0.143		

Figures are standardized regression coefficients (Beta) and 95% confidence intervals (CI) and coefficients of determination (R^2^). Adjusted for child’s age, age at arrival to Finland and mother’s SES

**Table 4:** Interaction of total CBCL symptoms and maternal depression, adjusted for child’s age, age at arrival to Finland and mother’s SES

Predictors	CBCL internal (child)			CBCL external (child)			CBCL total (child)		
	Beta	95% CI	*p*	Beta	95% CI	*p*	Beta	95% CI	*p*
Depression (mother)	− 0.03	− 0.16 to 0.10	0.703	− 0.10	− 0.23 to 0.02	0.119	− 0.07	− 0.20 to 0.06	0.303
CBCL total (parent)	0.27	0.15 to 0.40	** < 0.001**	0.38	0.26 to 0.50	** < 0.001**	0.33	0.21 to 0.45	** < 0.001**
Depression (mother) × CBCL total (parent)	− 0.20	− 0.34 to − 0.06	**0.005**	− 0.18	− 0.31 to − 0.04	**0.010**	− 0.23	− 0.36 to − 0.09	**0.001**
Observations	222			222			222		
R^2^/R^2^ adjusted	0.179/0.144			0.213/0.180			0.201/0.167		

Figures are standardized regression coefficients (Beta) and 95% confidence intervals (CI) and coefficients of determination (R^2^)

**Table 5:** Interaction of CBCL externalizing symptoms and maternal depression, adjusted for child’s age, age at arrival to Finland and mother’s SE

Predictors	CBCL internal (child)			CBCL external (child)			CBCL total (child)		
	Beta	95% CI	*p*	Beta	95% CI	*p*	Beta	95% CI	*p*
Boys									
Depression (mother)	− 0.11	− 0.33 to 0.10	0.363	− 0.26	− 0.42 to − 0.09	**0.002**	− 0.22	− 0.41 to − 0.03	**0.043**
CBCL external (parent)	0.40	0.20 to 0.60	** < 0.001**	0.72	0.57 to 0.87	** < 0.001**	0.53	0.34 to 0.71	** < 0.001**
Depression (mother) × CBCL external (parent)	− 0.06	− 0.27 to 0.16	0.614	0.05	− 0.12 to 0.21	0.565	− 0.07	− 0.27 to 0.13	0.486
Observations	98			98			98		
R^2^/R^2^ adjusted	0.199/0.127			0.539/0.498			0.344/0.285		
Girls									
Depression (mother)	− 0.08	− 0.27 to 0.11	0.187	− 0.12	− 0.29 to 0.06	0.073	− 0.10	− 0.28 to 0.08	0.076
CBCL external (parent)	0.05	− 0.13 to 0.23	0.498	0.28	0.11 to 0.45	**0.001**	0.13	− 0.04 to 0.31	0.107
Depression (mother) × CBCL external (parent)	− 0.28	− 0.52 to − 0.03	**0.028**	− 0.28	− 0.51 to − 0.05	**0.017**	− 0.34	− 0.58 to − 0.11	**0.005**
Observations	124			124			124		
R^2^/R^2^ adjusted	0.087/0.024			0.188/0.131			0.156/0.097		

Figures are standardized regression coefficients (Beta) and 95% confidence intervals (CI) and coefficients of determination (R^2^)

## Discussion

As in previous studies, our research revealed a discrepancy in reporting emotional problems of children also in this group of adopted children and their parents who have no shared genetic background. Our study showed that maternal depressive symptoms were related with poor agreement on reports for the total CBCL score, internalizing symptoms and for girls for externalizing symptoms too and thus supports the depression-distortion hypothesis.

It has been argued, that the nature of discrepancies differs by type of behavior. Some studies have found that there is higher agreement for externalizing problems, such as delinquent, aggressive, and antisocial behavior, than internalizing problems, such as withdrawal, anxiety and depression [41, 44, 48]. This might be due to the fact that internalizing problems may be harder to observe [27] and externalizing problems may be more obvious, more consistent across situations, or more persistent [48]. Moreover, some researchers have observed that parents tend to report more externalizing problems than adolescents [6, 11], and others, the converse [5, 42] or no difference [24]. Interestingly, some studies have found that parental depression is associated with higher agreement [30, 38] and that parents with psychopathology may be more accurate reporters due to their awareness of and sensitivity to mental health symptoms [25]. It might be argued, that mothers with depressive symptoms could be more accurate in their reports than those without.

Furthermore, Hughes and Gullone state that such discrepancies cannot indicate which informant is more accurate, or whether informants are over- or under-reporting the child’s behavior [25]. Rather, it could be argued that each informant provides unique information reflecting subjective, partial truths based on how and where they observe the behavior [6, 16].

The present study integrates and extends prior research on cross-informant discrepancy by using an adoption design to disentangle the contribution of genetic influences. A comparable study design was used by Tarren-Sweeney et al., who conducted a study on interrater agreement between foster parents and teachers [45]. They concluded that teachers and foster parents demonstrated moderate to good agreement (kappa = 0.70–0.79) in identifying clinically significant total problems and externalizing problems, but poor agreement in identifying internalizing problems. However, their study did not address the potential discrepancy between the children and foster parents. Because of this difference, the two studies are not fully comparable.

The finding that mothers with depressive symptoms and adopted girls report differently on externalizing symptoms is worth noticing. In the general population, females are considered at heightened risk for internalizing symptoms and males for externalizing symptoms. Watson et al. state that in children of depressed parents these normative gender differences may be even more evident, meaning that girls may be at even greater risk for internalizing problems and boys for externalizing problems [51]. Hence, the question arises, whether mothers with depressive symptoms are less tolerant and/or more sensitive to girls’ externalizing symptoms.

Another interesting aspect on parent–child discrepancy is how much and what kind of information it can reflect about the relationship between parents and children, especially in adoptive families. For instance, the question of the relevance of attachment constructs arises. Attachment itself can be considered to be related to the length of time the child has been living with the adoptive family.

## Strengths and limitations

The results of our study must be considered in the light of the study’s strengths and its limitations.

The major limitation was the cross-sectional design of our study. A longitudinal study would provide an opportunity to examine the stability and changes in maternal depressive symptoms and reporting of child behavioral problems by parents.

A considerable limitation of our study is the low number (7) of mothers who had depressive symptoms. Also, the mothers did not suffer from clinical depression but only had mild symptoms. It should also be taken into consideration that a number of studies have shown that internationally adopted children have more internalizing symptoms than their non-adopted peers [26].

Furthermore, adoptive parents have a lower threshold for referring their adolescents to treatment than biological parents, indicating that they might be more sensitive to potential problems and perhaps overestimate their occurrence [4]. Adoptive parents may also be more willing to seek help from a mental health professional for their troubled child because they are better educated or have greater economic resources than many non-adoptive parents have or because they have previously interacted with social service providers in the process of adopting. No prenatal information about the children and the life-events they experienced (except for the type and number of placements) before the moment they were adopted was available.

## Conclusions

The results of our research support the depression-distortion hypothesis in a study sample of genetically unrelated children and mothers, as maternal depressive symptoms were associated with less similar symptom reporting of child internalizing symptoms and girls’ externalizing symptoms compared to the children themselves.

It may be stated that clinicians and studies assessing children’s psychopathology should take into account parental current mood state. Furthermore, it would be of informative value to determine factors that influence agreement and discrepancies between informants. For instance, differences in parenting stress might be involved, especially when there is concurrent depression.

## Data Availability

The datasets during and/or analysed during the current study available from the corresponding author on reasonable request.
